# Exploring the Characteristics of Monkeypox-Related Genes in Pan-Cancer

**DOI:** 10.3390/cells11233909

**Published:** 2022-12-02

**Authors:** Yong Liao, Zhiping Liu, Weile Ye, Zunnan Huang, Jiaojiao Wang

**Affiliations:** 1 Center of Scientific Research, Maoming People’s Hospital, Maoming 525000, China; 2 Key Laboratory of Big Data Mining and Precision Drug Design of Guangdong Medical University, Key Laboratory of Computer-Aided Drug Design of Dongguan City, Key Laboratory for Research and Development of Natural Drugs of Guangdong Province, Guangdong Medical University, Dongguan 523808, China; 3 Guangdong Province Key Laboratory of Pharmacodynamic Constituents of TCM and New Drugs Research, College of Pharmacy, Jinan University, Guangzhou 510000, China

**Keywords:** monkeypox, cancers, immunity, genomics, methylation, prognosis

## Abstract

Monkeypox, an infectious virus that is a member of the Poxviridae family, has raised great threats to humans. Compared to the known oncoviruses, the relationship between monkeypox and cancer still remains obscure. Hence, in this study, we analyzed the multi-omics data from the Cancer Genome Atlas (TCGA) database by using genomic and transcriptomic approaches to comprehensively assess the monkeypox-related genes (MRGs) in tumor samples from 33 types of cancers. Based on the results, the expression of MRGs was highly correlated with the immune infiltration and could be further utilized to predict survival in cancer patients. Furthermore, it was shown that tumorigenesis and patient survival were frequently associated with the genomic alterations of MRGs. Moreover, pathway analysis showed that MRGs participated in the regulation of apoptosis, cell cycle, Epithelial to Mesenchymal Transition (EMT), DNA damage, and hormone androgen receptor (AR), as well as RAS/MAPK and RTK signaling pathways. Besides, we also developed the prognostic features and consensus clustering clusters of MRGs in cancers. Lastly, by mining the cancer drug sensitivity genomics database, we further identified a series of candidate drugs that may target MRGs. Collectively, this study revealed genomic alterations and clinical features of MRGs, which may provide new hints to explore the potential molecular mechanisms between viruses and cancers as well as to provide new clinical guidance of cancer patients who also face the threats during the monkeypox epidemic.

## 1. Introduction

According to the International Agency for Research on Cancer (IARC) of World Health Organization (WHO), there were an estimated 19.3 million new cancer cases and nearly 10 million cancer deaths worldwide by 2020, and the global cancer burden was projected to reach 28.4 million cases by 2040, a 47% increase from 2020; therefore, the development of cancer control measures is critical for global cancer control [1].

Previous studies have shown that some kinds of viruses play specific roles in tumorigenesis and progression [2]. For example, Herpes simplex virus type-1 (HSV-1) infection promoted the growth of uveal melanoma cultures [3]. Besides, the Kaposi’s sarcoma-associated herpesvirus (KSHV) is identified as one of the causative agents of Kaposi’s sarcoma, primary exudative lymphoma, and multicentric Castleman disease [4]. Moreover, Epstein–Barr virus (EBV) is found to be linked with Burkitt’s lymphoma, Hodgkin’s lymphoma, post-transplant lymphoma, and nasopharyngeal carcinoma [5,6,7]. Given the association between the listed viruses and cancers, it is intriguing to explore whether monkeypox and tumors are also related as the monkeypox outbreak has become a public health emergency across multiple counties.

Monkeypox is caused by the monkeypox virus from the Poxviridae family. As an emerging zoonotic pox virus, it is continuing to infect humans as well as wild and domestic animals and remains one of the greatest threats to human and animal health [6,7]. Recently, about 3000 new cases of monkeypox infection have been reported in U.S. [8]. Furthermore, a gene expression profiling of monkeypox virus-infected cells showed that the monkeypox infection may be involved in the tumor-related signaling pathways [9]. However, the studies in the field of tumor virology remains rocky, and it is still urgent to explore the relationship between monkeypox and tumors [10].

Advances in clinical studies of viral therapies suggest that emerging viral-targeted cancer strategies are worthy of being promising additions to conventional therapies. However, changes and interactions in solid tumors following viral therapy are complicated and may be influenced by multiple factors [11]. The relevance of monkeypox to tumors has not been studied to date, and the role of monkeypox in cancers remains largely unknown. A few questions need to be answered first. Which signaling pathways may be affected by monkeypox infection in tumors? What are the upstream regulators of these pathways? Is monkeypox involved in the regulation of immune infiltration? What is the impact of monkeypox on the molecular mechanism of oncology drug actions? What is the possibility of monkeypox as a bio-predictive marker of prognosis in tumors? Those unsolved questions derived from this topic deserve further exploration.

In this study, we comprehensively evaluated the genomic and clinical characteristics of monkeypox-related genes in th33 tumors and explored the relationship between monkeypox-related genes and tumor immune infiltration, tumor-associated pathways, and drug sensitivity. Monkeypox-related genes (MRGs) in tumors were also utilized for consensus clustering grouping and the construction of prognostic predictive features. As the first exploration of the relationship between monkeypox and pan-tumor, this study provides a valuable resource for tumor virus research and tumor-targeted therapy.

## 2. Materials and Methods

### 2.1. Cell Lines and qRT-PCR

HK-2, 786-O, ACHN, and Caki-1 cell lines were obtained from American Type Culture Collection^®^ (ATCC, Manassas, VA, USA) and subsequently cultured following ATCC handling information [12].

Cells were seeded in a 6-well plate with a density of 5 × 10^4^. When the cells were confluent, total RNA of cell cultures was collected using Trizol reagent (Invitrogen, New York, NY, USA) and extracted according to the manufacturer’s instructions. The cDNA was made using iScript cDNA synthesis kit (Bio Rad, Hercules, CA, USA). Real-time PCR analysis was performed with Power SYBR Green PCR Master Mix (Thermo Fisher Scientific, Waltham, MA, USA) with the respective gene-specific primers listed in Table 1 by a qPCR system (LightCycler^®^ 480, Roche Life Science). β-actin was used as an internal control. The relative difference was expressed as the fold-matched control values calculated with the efficiency-corrected 2^−ΔΔCT^ method [13,14]. The primer list for quantitative RT-PCR is provided in Table 1.

### 2.2. Tumor Types

Tumor-related data utilized in this study included mRNA Seq data (n = 10,471), clinical data (n = 11,160), single nucleotide variant (SNV) data (n = 10,234), copy number variant (CNV) data (n = 11,461), and methylation data (n = 10,063), all of which were from The Cancer Genome Atlas (TCGA) (https://portal.gdc.cancer.gov/ (accessed on 12 May 2022)) [15]. Data collection relied on the GSCA database (http://bioinfo.life.hust.edu.cn/GSCA (accessed on 12 May 2022)) [16]. Reverse phase protein array (RPPA) data for pathway studies was obtained from The Cancer Proteome Atlas (TCPA) (https://tcpaportal.org/tcpa/index.html (accessed on 13 May 2022)) [17]. Immunogenomic analysis was performed by the ImmuCellAI algorithm on 24 immune cells [18]. The correlation between gene expression and drug sensitivity was investigated using the Genomics of Drug Sensitivity in Cancer (GDSC) database (www.cancerrxgene.org (accessed on 13 May 2022)) [19]. MRGs (inclusion criteria were mRNA) were queried using the GeneCards database (https://www.genecards.org/ (accessed on 10 May 2022)) [20]. The abbreviation of the cancer types is listed below (Table 2).

### 2.3. Comparison of GSVA Scores between Tumor and Normal Samples

A particular cancer sample population’s changes in MRGs activity (expressed as GSVA scores) were evaluated by unsupervised method. Then, analysis of differential gene set activity between tumor and normal samples was performed. GSVA scoring is mainly done by transforming the expression matrix of genes between samples into the expression matrix of gene sets between samples. Next, changes in gene set activity (denoted GSVA score) for a specific cancer sample group were also estimated in an unsupervised manner. The GSVA score of MRGs (MRGScore) represents the combined level of monkeypox-associated gene. The GSVA score was calculated by the R package GSA [21].

### 2.4. GSVA Scores and Survival

In order to analyze survival of MRGs and tumor, the association between MRGScore and survival was assessed. Clinical data used in this study were from 33 different tumor samples, and uncensored data were not included. According to the median GSVA value, tumor samples were divided into groups with high and low GSVA scores. Following that, survival time and status of both groups were fitted using the R package survival. The risk of survival for each gene was determined by Cox proportional-hazards models and Log-rank tests.

### 2.5. GSVA Scores and Subtypes

Clinical data of tumor samples from 9 cancer types (HNSC, LUSC, COAD, STAD, LUAD, GBM, BRCA, KIRC, and BLCA) were used. At least five samples must be included in a subtype. The MRGScore was compared between groups with the Wilcoxon test (number of subtype groups = 2) and the ANOVA test (number of subtype groups > 2).

### 2.6. Relationship between GSVA and Tumor Stage Staging

Using the results from 4 types of stage (pathologic, clinical, masaoka (for THYM only), and IGCCCG stage (for TGCT only)) data of 9478 tumor samples from 27 cancer types (ACC, BLCA, BRCA, CESC, CHOL, COAD, DLBC, ESCA, HNSC, KICH, KIRC, KIRP, LIHC, LUAD, LUSC, MESO, OV, PAAD, READ, SKCM, STAD, TGCT, THCA, THYM, UCEC, UCS, and UVM) were used. GSVA score and clinical staging data were combined by sample barcoding. At least 5 samples from each carrier table subgroup were required. The Wilcoxon test (n = 2) and the ANOVA test (n = 3) were applied to compare GSVA scores between groups.

### 2.7. Correlation between GSVA Scores and Pathway Activity

RBN RPPA data were centered on the median and normalized by sample standard deviation to get relative protein levels. Pathway activity scores were then calculated by adding all positive regulatory protein levels and subtracting all negative regulatory protein levels [22]. In an unsupervised way, the GSVA score describes the gene set activity variation of a specific cancer sample population. Moreover, the correlation between GSVA score and pathway activity can be calculated. This suggests that pathway activity could be defined by the pathway activity score.

### 2.8. Relationship between GSVA Score and Immunization

The immune infiltration and GSVA scoring modules estimated the association between immune cell infiltration and gene set expression levels. Infiltration analyzed 24 immune cell infiltrations. The association between immune cell infiltration and gene set expression levels was expressed as a correlation coefficient and evaluated by Spearman’s correlation analysis. *p*-values were adjusted by FDR.

### 2.9. The Relationship between SNV and Survival

SNV data and clinical survival data were collected from the TCGA database and combined by sample barcodes. The gene set SNV indicates the integrated SNV status of the input gene set for each sample. Only when at least one gene from the input gene set is altered in a sample does this sample classify as a mutation set. The sample is categorized as a WT group if there are no SNVs in any of the genes in the input gene set. Regarding the mutant group, samples showing deleterious mutants were included in this study. Further survival analysis could be performed on the group that comprises more than 2 samples, and a minimum of 2 groups was needed. Two groups of survival and survival status were fitted using the R package survival. A Cox proportional-hazard model and a Log-rank test were used to test survival differences between the groups.

### 2.10. The Relationship between CNV and Survival

From the TCGA database, clinical survival and CNV data were retrieved. Next, merge the two datasets via bar-coding. Samples with competing cancer mortality risks were eliminated (for DSS and DFI data). Gene set CNV, like SNV gene sets, reflects the integrated CNV status of each sample’s input gene set. Only samples in which at least one gene from the input gene set was consistently amplified or deleted were grouped as Amp. or Dele. However, if no CNVs exist in any of the genes in the input gene, the sample is classified as a WT group. Genes with inconsistent CNV status, for example gene A being amplified and gene B being deleted in sample 1, would be excluded from this study. For survival analysis to be carried out, there have to be at least two groups with more than two samples. Groups with <2 samples were also retained to plot survival curves. The R package survival was used to fit survival time and status within the gene set CNV group. In addition, Log-rank tests were performed to compare group survival.

### 2.11. The Relationship between SNV and Immune Infiltration

ImmuCellAI was used to assess the infiltration of 24 different types of immune cells. Only if the input gene set contains at least one mutant gene can a sample be categorized as a mutation group. However, if there is no SNV in any of the genes in the sample, it will be classified as the WT group. The correlation between immune cell infiltration and gene-set SNV was assessed by comparing the mean infiltration between gene set CNV groups by the Wilcoxon test. *p*-values were adjusted by FDR.

### 2.12. Drug Sensitivity Analysis

We collected the IC50 and its corresponding mRNA gene expression for 265 small molecules in 860 cell lines from Genomics of Drug Sensitivity in Cancer (GDSC, https://www.cancerrxgene.org/ (accessed on 10 May 2022)). The data on mRNA expression and drug sensitivity were merged. A Pearson correlation analysis was conducted to determine the relationship between gene mRNA expression and medication IC50. *p*-values were adjusted by FDR. A positive association suggests that high gene expression indicates drug resistance, while a negative association predicts drug sensitivity.

### 2.13. MRG-Related Prognostic Prediction Feature Construction in UVM

To minimize the size of the gene set, a Least Absolute Shrinkage and Selection Operator (LASSO) analysis was performed. The minimal lambda value was defined as the optimal value [23]. The R software survival package was used to do multivariate Cox regression analysis and risk prognostic modeling [24,25]. This model is a RiskScore formula with numerous genes, each with a weight; negative values represent protected genes and positive numbers represent risk genes. Based on those genes’ median values, we divided patients into high-risk and low-risk categories. Log-rank was used to compare Kaplan–Meier (KM) survival between two or more groups. At the same time, we used the ROC methods to assess how efficiently the model could predict. For KM curves, *p*-values and hazard ratios (HR) with 95% confidence intervals (CI) were derived by the Log-rank test and univariate Cox regression.

### 2.14. Identification and Survival Assessment of Molecular Subpopulations

RNAseq data (Level 3) of the tumors and corresponding clinical information were obtained from The Cancer Genome Atlas (TCGA) dataset (https://portal.gdc.com (accessed on 10 May 2022)). Consistency analysis was performed using the R package ConsensusClusterPlus (v1.54.0) with a maximum number of clusters of 6 and 100 replicates to extract 80% of the total sample: cluster Alg = “chi” and inner Linkage = “ward. D2”. All of the clustering heat maps were analyzed by the R software package heatmap (v1.0.12). In all gene expression heat maps, genes with a variance of larger than 0.1 were taken out.

### 2.15. Functional Analysis

The “Limma” R package was used to identify differentially expressed genes (DEGs) between two clusters. Gene ontology (GO) analysis and Kyoto Encyclopedia of Genes and Genomes (KEGG) analyses were conducted using the “clusterProfiler” R package to find relevant pathways, which were then visualized in Metascape 5. Based on the “GO Bioprocess” gene set downloaded from the Molecular Signature Database (see text footnote 5), a Gene Set Variation Analysis (GSVA) was performed using the “GSVA” R package to demonstrate altered signaling pathways between two clusters. In addition, Gene Set Enrichment Analysis (GSEA) was performed to analyze the differences across clusters based on the same dataset.

### 2.16. Immunity Correlation Analysis of Subgroups

TIMER immune infiltration analysis was done to calculate the abundance of six immune infiltrating cells (covering B cells, macrophages, dendritic cells, neutrophils, CD4 T cells, and CD8 T cells) in various subgroups [26]. *SIGLEC15*, *TIGIT*, *CD274*, *HAVCR2*, *PDCD1*, *CTLA4*, *LAG3*, and *PDCD1LG2* are immunological checkpoint genes [27,28,29,30]. Hence, we extracted the expression values of these eight genes to observe the expression of immune checkpoint-related genes in the subgroups.

### 2.17. Statistical Analysis

Unless otherwise specified, all statistical analyses were performed using R software (v4.0.3). The Spearman correlation test and the Cox proportional risk models were respectively used for correlation analysis and calculation of survival risk and HR. Each variable’s prognostic significance was evaluated using Kaplan–Meier survival curves and compared using Log-rank tests. The rank sum test was conducted to differentiate between two datasets. *p* value of < 0.05 was deemed statistically significant, represented by a “*”. In addition, “**” and “***” reflect *p* values of less than 0.01 and less than 0.005, respectively. The data were extracted and analyzed until 20 May 2022. The operations of this study were all in accordance with public database regulations, and the above data were open access and did not require additional consent from the local ethics committee. The significance of the differences between two groups was assessed by using Mann–Whitney U test (n < 5). Multiple comparisons were performed by one-way ANOVA followed by Bonferroni’s post hoc test. All results are showed as mean ± SD. All biological experiments were repeated three times using independent cell cultures (biological replications). *p* < 0.05 was considered significant.* *p* < 0.05, ** *p* < 0.01, *** *p* < 0.001, **** *p* < 0.001.

## 3. Results

### 3.1. Difference of MRGs between Tumor and Normal Tissues

“Monkeypox” was entered into the GeneCards database to find Monkeypox-related genes (MRGs), which are listed in Table 3 below.

**Figure 1:**
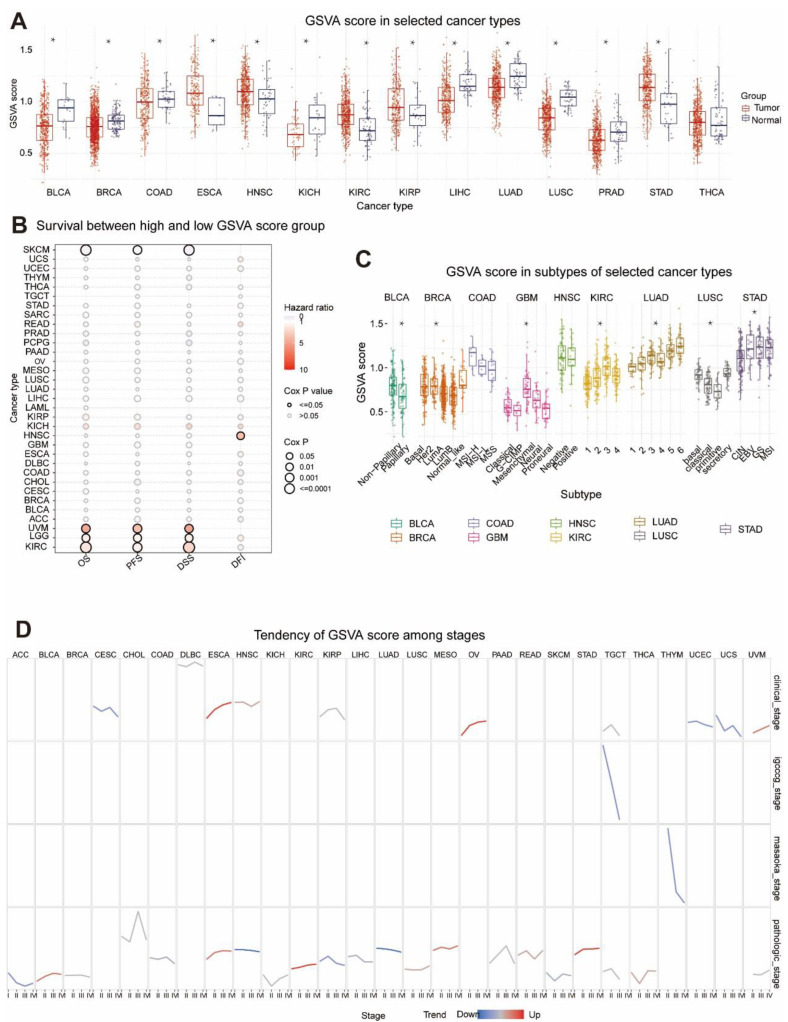
Expression, survival, subtype, and staging analysis of GSVA scores for MRGs in pancytopenia. (**A**) Box plots comparing GSVA scores of tumor and normal samples. *p*-values: statistical significance estimated by a *t*-test. * *p*-value < 0.05, significant difference. (**B**) Results of survival differences between GSVA score groups in cancer. Risk ratios and Cox*p* values are shown by bubble color and size. The row is the type of survival, and the column is the selected cancer type. Bubble colors from blue to red represent low- to high-risk ratios; bubble size is positively correlated with Cox*p*-value significance. Black outlined borders of bubbles indicate Cox*p* values ≤ 0.05. (**C**) Box plot showing GSVA scores for subtypes of selected cancers. *p*-values: statistical significance estimated by Wilcoxon test (subtype group = 2) and ANOVA test (subtype group > 2). * *p*-value < 0.05, a significant difference. (**D**) Presentation of GSVA score trends from stage I to stage IV. The trend line colors of blue and red indicate decreasing and increasing trends, respectively.

GSVA scores of MRGs were firstly calculated to investigate their influence on various cancer types in clinical trials. As illustrated in Figure 1, MRGs expression vs. survival, cancer subtype, and staging were analyzed based on the GSVA scores. In 13 solid tumors, except THCA, GSVA scores were significantly different from neighboring normal tissues (*p* < 0.05), which indicates that MRGs were expressed differently between tumors and normal tissues and that they may be involved in tumor initiation and progression. Furthermore, elevated GSVA scores in MRGs were positively associated with poor survival in UVM, LGG, KIRC, and HNSC (Figure 1B), suggesting that MRGs might be the risk factor of these cancers. Notably, among the listed cancer types, UVM was shown to have the greatest risk ratio and risk factor predilection based on the GSVA score (Cox*p* < 0.05).

Figure 1C shows that GSVA scores of MRGs in BLCA, BRCA, GBM, KIRC, LUAD, LUSC, and STAD were significantly linked with tumor subtype. In contrast, there was no differential expression of GSVA scores in early COAD and HNSC, indicating no correlation between them (*p* < 0.05). In the study of clinical phases, GSVA scores of CESC, UCEC, and UCS tended to go down, while ESCA and OV scores went up (Figure 1D). There was a downward trend in GSVA scores in both the germ cell tumor stage (designated TGCT) and Masaoka stage (THYM only). Pathological staging analysis revealed a decreasing trend in GSVA scores for the ACC, HNSC, KIRP, and LUAD and a rising trend in GSVA scores for the BLCA, ESCA, KIRC, MESO, and STAD. Based on these results, it seemed that the expression of MRGs is strongly linked to the clinical and pathological staging of tumors, and that their abnormal expression may be linked to the growth of tumors.

### 3.2. Relationship between GSVA Score and Immune Infiltration

**Figure 2:**
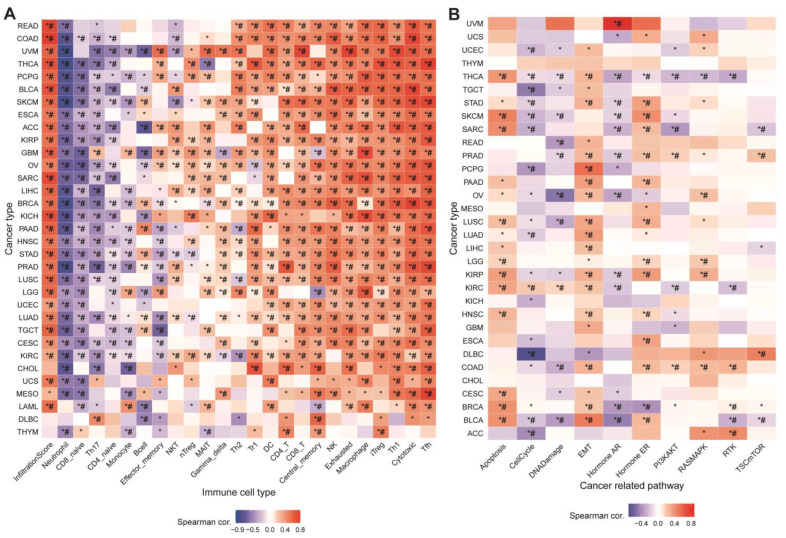
Correlation analysis of GSVA scores of MRGs with immune cells and cancer-related pathways in pan-cancer. * *p* value <= 0.05; # FDR <= 0.05. (**A**) Heat map summarized the analysis of Pearman correlation between GSVA scores of MRGs and immune cell infiltration. (**B**) Correlation between GSVA score and pathway activity in different cancer types.

Immune infiltration generally modifies the tumor microenvironment, which further affects tumor growth and invasion [31]. Calculating the GSVA and infiltration scores revealed that both were positively associated with most cancers (Figure 2A). In addition, the correlation was showing positively in most cases, with the exception of negative correlations, specifically in neutrophils, CD8-naive cells, Th17 cells, B cells, and effector memory cells. Upregulation of MRGScore probably increased immune infiltration and immune cell abundance, which altered tumor progression, demonstrating that MRGs are strongly connected with the tumor immune microenvironment.

### 3.3. Association between MRGs and Cancer-Related Pathways

We analyzed the association between MRG GSVA scores and cancer-related pathway networks to better understand the specific pathways or biological processes by which MRGs affect tumors. Figure 2B showed that MRGs were significantly involved in 10 cancer-related signaling pathways, including apoptosis, cell cycle, DNA damage, EMT, and others. In the majority of tumors, MRGs’ GSVA scores were positively correlated with apoptosis, EMT, hormone ER, and RAS/MAPK, while being negatively associated with the cell cycle, DNA damage, hormone AR, and PI3K/AKT. The GSVA scores of MRGs revealed linkage in different pathways as well as various roles in multiple pathways. These data imply that MRGs regulate cancer-related pathways and that monkeypox may affect tumor growth by activating or inhibiting these pathways.

### 3.4. The Relationship between Tumor Survival and MRGs’ SNV and CNV

**Figure 3:**
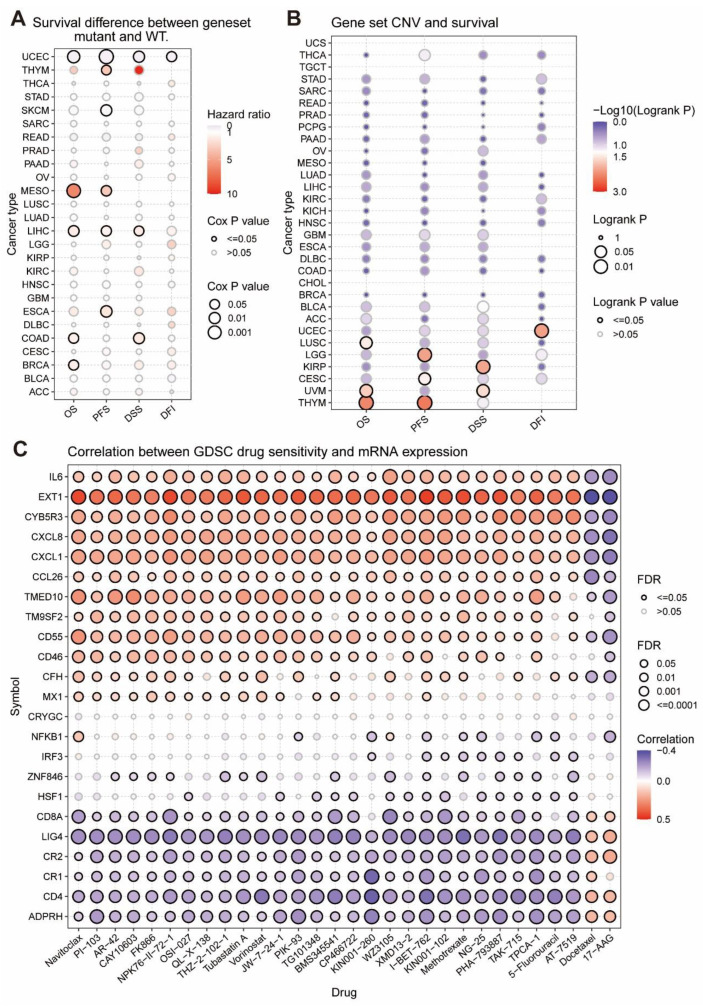
Relationship between MRGs and tumor survival as well as tumor drug resistance. (**A**) Risk ratios and Cox*p* values are shown by bubble color and size. The column indicates cancer type, and the rows denote survival type (OS, PFS, DSS, and DFI). Bubble color from blue to red indicates low- to high-risk ratios, and bubble size is positively correlated with Cox*p* value significance. Black outline borders indicate Cox*p* values ≤ 0.05. (**B**) Log-rank *p* values are shown by bubble color and size. The column selects the cancer type, and behavioral survival type (OS, PFS, DSS, and DFI). Bubble colors, from blue to red, indicate low- to high-risk ratios, and bubble size is positively correlated with the significance of Log-rank *p* values. Black outline borders indicate Log-rank *p* values ≤ 0.05. (**C**) Bubble plots to summarize the correlation between input genes and drugs. Only when genes associated with at least one drug were obtained. In addition, only when drugs associated with at least one gene were obtained. Blue bubbles indicate negative correlations, while red bubbles indicate positive correlations. The darker the color of these bubbles, the higher the correlation. Bubble size is positively correlated with FDR significance. Black outline borders indicate FDR ≤ 0.05. Plots for the top 30 drugs are shown. These drugs are ranked by the correlation coefficient of the searched genes and the combined level of FDR.

Genetic mutations including copy-number variants (CNVs) and single nucleotide variants (SNV) contribute to cancer heterogeneity and challenges to cancer treatment [32]. Thus, in Figure 3A, it illustrated that, in UCEC and SKCM, the HR of SNV scores of MRGs was less than 0, and mutations in MRGs were associated with high survival and were protective factors. However, in THYM, MESO, LIHC, ESCA, COAD, and BRCA, the HR of the SNV fraction of MRGs was greater than 0, and mutations in MRGs were associated with low survival and were risk factors. As was demonstrated in Figure 3B, CNV pooled scores of MRGs in UEC, LUSC, LGG, KIRP, CESC, UVM, and THYM were positively associated with survival, while they were not negatively correlated in other tumor samples and were generally unrelated, implying that MRGs’ CNV is a protective factor that may promote good prognosis.

### 3.5. The Considerable Impact of MRGs on the Drug Sensitivity of Tumors

Genomic changes affect the clinical response of patients to drugs. We combined a drug sensitivity study of GDSC cancer cell lines to examine MRGs’ influence on tumor drug resistance. Figure 3C indicates a positive correlation between the expression of the eight genes *ADPRH*, *CD4*, *CR1*, *CR2*, *LIG4*, *CD8A*, *HSF1*, and *ZNF846* and the IC50 of 28 medications; that is, it shows a positive relationship between the expression of the eight genes and tumor resistance to the treatments. Moreover, the expression of 11 genes (namely *MX1*, *CFH*, *CD46*, *CD55*, *TM9SF2*, *TMED10*, *CCL26*, *CXCL1*, *CXCL8*, *CYB5R3*, *EXT1*, and *IL6*) was negatively correlated with the IC50 of 28 drugs in tumors, i.e., the tumor resistance to these drugs was negatively correlated with the expression of these genes. High gene expression may boost these drugs’ capacity to cure cancer. According to these findings, the differential expression of MRGs may influence how well medications work against cancers by correlating with tumor resistance to drug treatment.

### 3.6. MRGs-Related Prognostic Prediction Feature Construction in UVM

**Figure 4:**
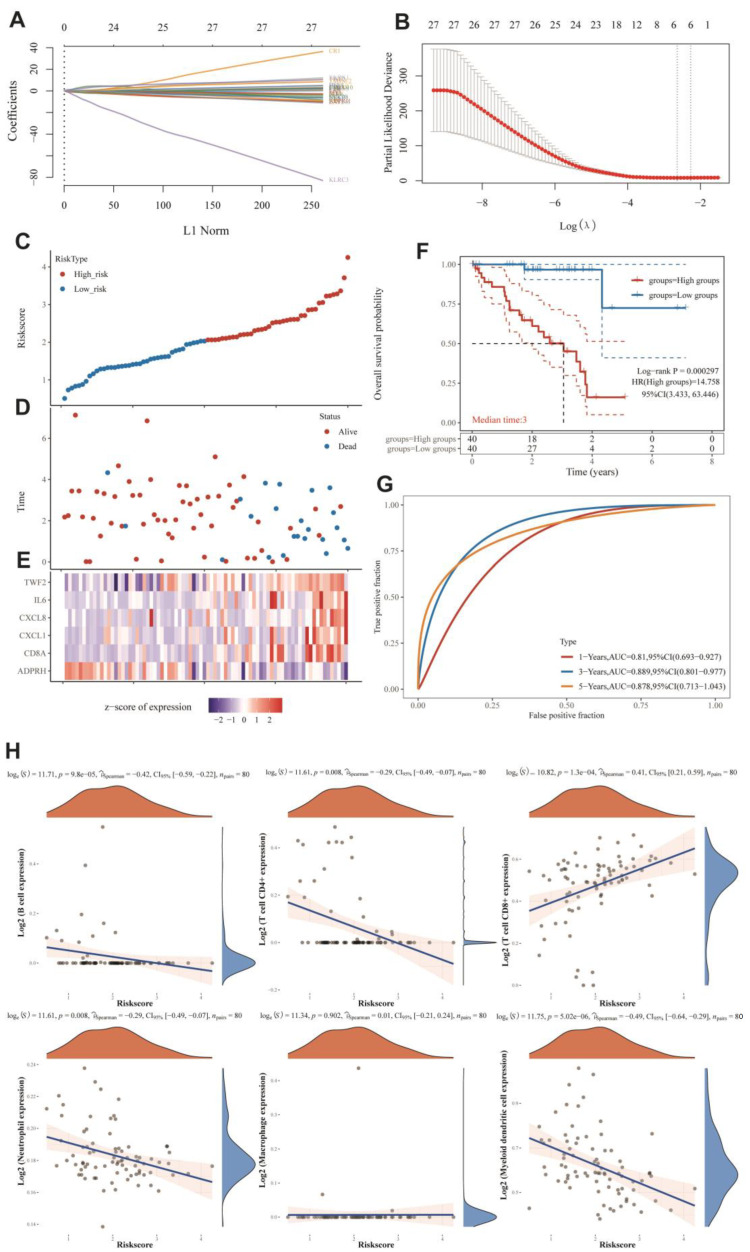
Prognostic risk model of MRGs in UVM and its analysis with immune cell correlation. (**A**) LASSO coefficients of MRGs were plotted at the values chosen for 10-fold cross-validation. (**B**) Cross-validation error curves for the selected tuning parameter (log λ). Vertical dashed lines were plotted at the optimal values. (**C**) Sample distribution of risk score analysis based on the prognostic risk model of MRGs. (**D**) Different patterns of survival status and survival time for high- and low-risk samples. (**E**) Heat map of cluster analysis showed the expression of MRGs of the risk model in each patient. (**F**) Kaplan–Meier survival curves for OS of patients in the high-risk and low-risk groups. If HR > 1, it means the model is a risk model; if HR < 1, it means the model is a protection model; 95% CL represents the HR confidence interval; median time represents the time (i.e., median survival time) corresponding to the survival rate at 50% in both high-risk and low-risk groups, in years. (**G**) The ROC curves of this risk model at different times with AUC, where higher AUC values indicate the stronger predictive ability of the model. (**H**) Heat map of correlation between prognostic risk model and immune score, where both horizontal and vertical coordinates represent genes, different colors represent correlation coefficients, and darker colors represent the stronger correlation between the two.

The relationship between UVM and monkeypox has not been reported yet. Furthermore, the MRGs were shown to be differentially expressed in UVM based on our previous analyses and have the highest prognostic HRs with UVM. Thus, we continued to investigate the survival predictive role of MRGs in UVM. In Figure 4A,B, we used multivariate Cox analysis based on Lasso regression to construct a risk model to obtain more accurate predictive characteristics:

*Riskscore* = (*−0.6917*) ∗ *ADPRH* + (*0.0858*) ∗ *CD8A* + (*0.1109*) ∗ *CXCL1* + (*0.2899*) ∗ *CXCL8* + (*0.3011*) ∗ *IL6* + (*0.8819*) ∗ *TWF2*)

**Figure 5:**
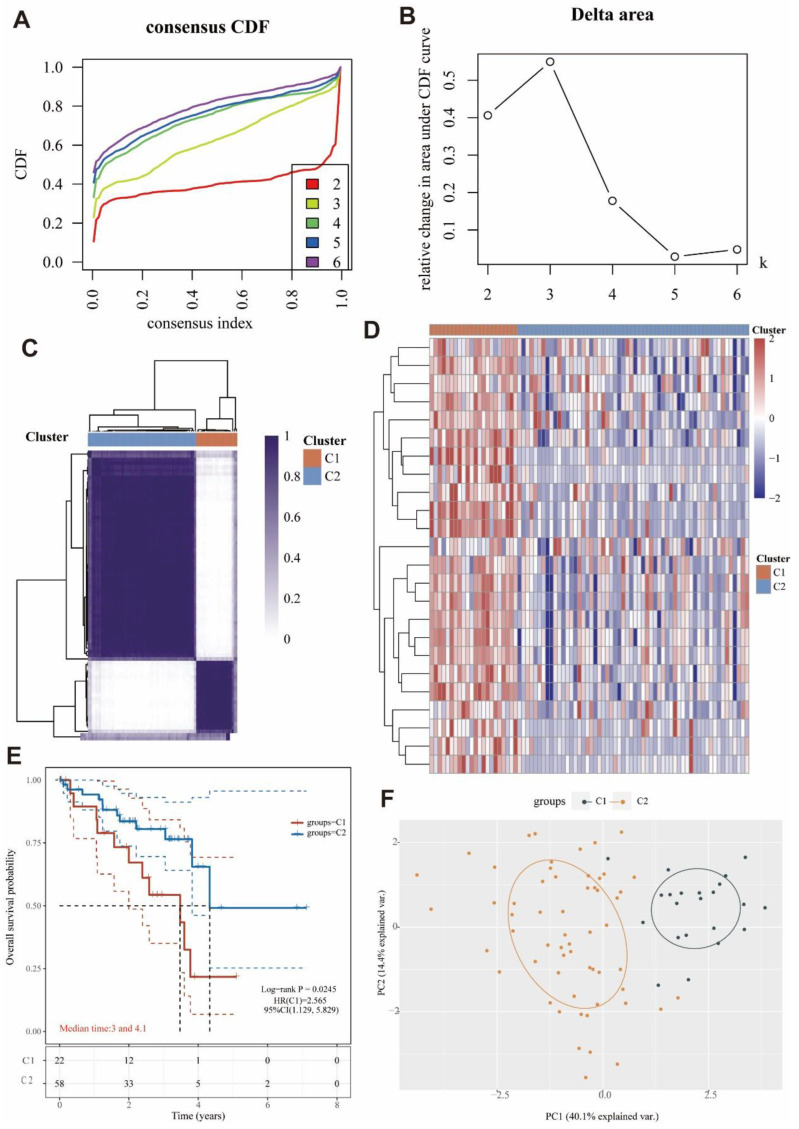
Subgroups typing of UVM by MRGs (**A**) The colors of CDF curves represent the number of different subgroups in the grouping. (**B**) CDF Delta area curves. Delta area curves for consistent clustering indicate the relative change in the area under the cumulative distribution function (CDF) curve for each category number k compared to k-1. The horizontal coordinate indicates the category number k, and the vertical coordinate indicates the relative change in the area under the CDF curve. (**C**) Consensus clustering matrix for k = 2. (**D**) Heat map features of the two clusters of cluster 1 and 2 (C1 and C2). (**E**) KM survival curves of different clusters of samples in the dataset, where different groups were tested by Log-rank, and 95% CL represents the HR confidence interval. Median time represents the time corresponding to the survival rate of different groups at 50% (i.e., median survival time), in years. (**F**) Scatter plot of the sample distribution.

In this part, we show that the established risk model successfully classified MESO patients into high-risk and low-risk groups. Figure 5D shows that low-risk groups had more survival samples than high-risk groups. We also observed that *TWF2*, *IL6*, *CXCL8*, *CXCL1*, and *CD8A* tended to be highly expressed in the high-risk group, while the tendency of ADPRH was to be highly expressed in the low-risk group (Figure 4E). Figure 4F demonstrates that the low-risk group had a longer overall survival rate than the high-risk group. Figure 4G showed that the constructed risk model had good predictive performance with an area under the curve (AUC) of 0.81, 0.889, and 0.878 for the 1-year, 3-year, and 5-year ROC curves, respectively.

### 3.7. Immuno-Correlation Analysis of Prognostic Features of MRGs

Given the significant correlation between MRGs and immunity in pan-cancer, we further analyzed the immune correlation of MRGs prognostic features in UVM. Figure 4H showed that the prognostic characteristics of MRGs were positively correlated with the expression of B cells, CD4^+^ T cells, neutrophils, and myeloid dendritic cells; on the contrary, they were negatively correlated with the expression of CD8^+^ T cells. These results suggested that in UVM, MRGs’ prognostic features were highly correlated with the immune microenvironment and had diverse effects on immune cells. It implies that MRGs may have opposite roles on the regulation of different kinds of immune cells.

### 3.8. Identification of Two Molecular Subtypes Based on MRGs

By using a consensus clustering approach, the UVM patients in the cohort were divided into two clusters (C1 and C2) based on the expression of MRGs (Figure 5A–C). Overall, 22 patients were clustered into C1, and 58 patients were clustered into C2. The expression levels of MRGs in both clusters were demonstrated by a heat map (Figure 5D). The significant difference of the expression was found between C1 and C2, while the MRGs were more expressed in C1 than C2. In addition, overall survival was higher in C2 patients than in C1 patients (*p* = 0.0245; Figure 5E). The high expression of MRGs may be associated with the low survival in C1. In Figure 5F, we also could see that the genes were clearly stratified into two clusters. These results suggested that MRGs were able to classify osteosarcoma patients into two molecular subtypes with different overall survival.

### 3.9. DEGs and Functional Analysis

**Figure 6:**
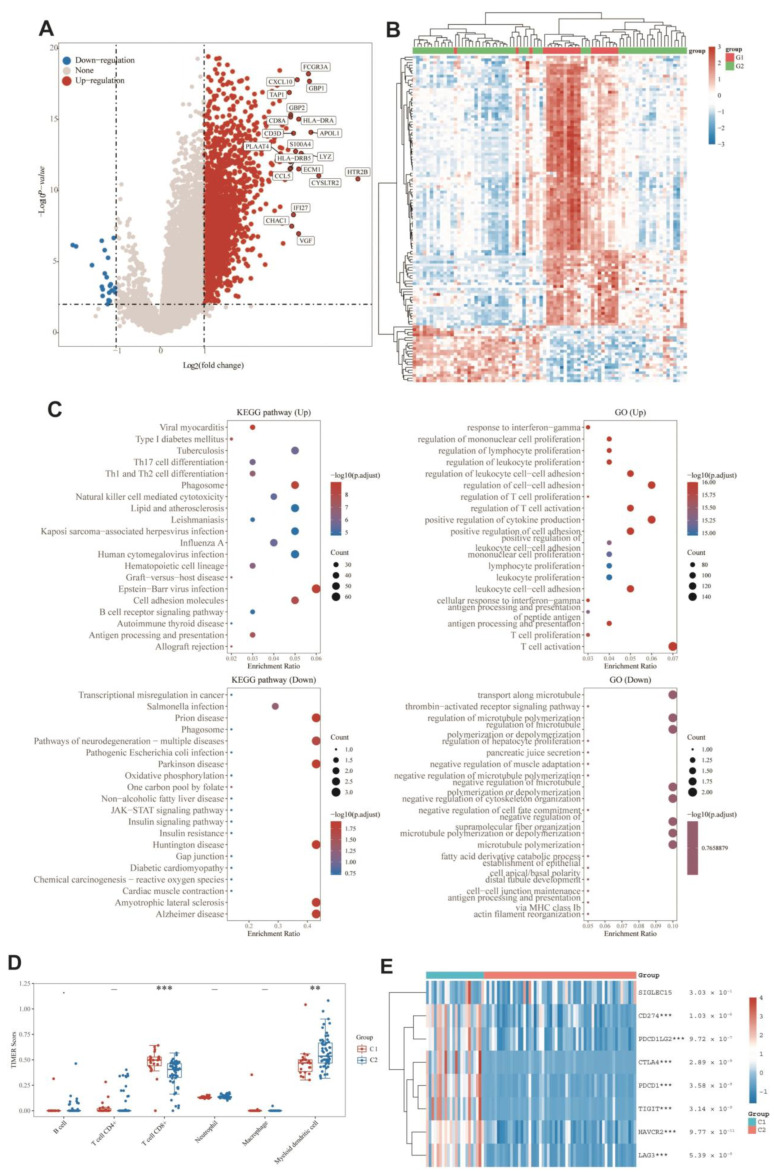
Identification of differential genes between clusters and functional analysis. (**A**) Volcano plotting with fold change and corrected *p*-values. In the diagram, red dots indicate genes that are significantly differentially upregulated, blue dots represent genes that are significantly differentially downregulated, and gray dots denote genes that are not significant. (**B**) Heat map of differential gene expression, where different colors represent the expression trends in different tissues. Due to the large number of differential genes, the 50 most differentially altered upregulated genes and 50 downregulated genes are shown here. Figure 6A,B showed that a total of 2412 DEGs were detected, of which 22 genes were downregulated and 2390 genes were upregulated in C1. (**C**) The results of differential upregulated genes KEGG pathway enrichment, differential upregulated genes GO term enrichment, differential downregulated genes KEGG pathway enrichment, and differential downregulated genes GO term enrichment. The different colors represent the significance of the differential enrichment results, and the larger the value, the smaller the FDR value. The size of the circle represents the number of enriched gene, and the larger the number, the larger the circle. Enrichment results with *p* value < 0.05 or FDR < 0.05 are considered to be enriched to a significant pathway, i.e., the right scale of the enrichment graph -log10 (*p* value) is greater than 1.3. (**D**) Distribution of TIMER immune scores in the two clusters, where the horizontal coordinate represents the type of immune cell infiltrating cells and the vertical coordinate represents the distribution of this immune infiltration score in different groups, ** *p* value < 0.01, *** *p* value < 0.001, and the asterisk represents the degree of significance. Significance was tested by the Wilcox test. (**E**) Heat map of expression of immune checkpoint-associated genes in two clusters, where different colors represent the expression trends in different samples. ** *p* value < 0.01, *** *p* value < 0.001, and asterisks represent the level of significance. Significance was tested by the Wilcox test.

DEGs between the two groups were identified, and functional analysis was performed to explore potential signaling mechanisms. Figure 6A,B showed that a total of 2412 DEGs were detected, of which 22 genes were downregulated and 2390 genes were upregulated in C1. KEGG pathway analysis (Figure 6C) showed that C1 was related with the elevation of post-infection emergency immune pathways, such as viral infection, B-cell pathway, and cytotoxicity, and downregulated in disease pathways such as neurodegeneration, Parkinson’s, and Huntington’s. As for GO enrichment analysis (Figure 6C), the upregulated DEGs were enriched in immune-related biological processes such as T-cell activation, T-cell proliferation, antigen processing and presentation, immune cell differentiation, and leukocyte proliferation. Similarly, GO enrichment analysis also identified downregulation in microtubule-related pathways such as microtubule transport and microtubule regulation. Cluster differences were strongly correlated with immunity, suggesting that immunity may be related to the role of monkeypox on bone UVM. Next, the immunoassays were performed to explore the immune differences between the two molecular subtypes. When UVM patients with C2 were compared to those with C1, the TIMER algorithm disclosed that CD8^+^ cell expression was lower and dendritic cell expression was higher (Figure 6D). No other immune cells were detected in a way that was statistically significant. Furthermore, as seen by the heat map in Figure 6E, the expression of immune checkpoint genes was significantly different between C1 and C2, with lower expression in C2 indicating a relatively higher C2 immune status. On the basis of these findings, it seems that there is a significant difference in immune status between the two molecular subtypes.

### 3.10. Confirmation of MRGs Expression in KIRC

**Figure 7:**
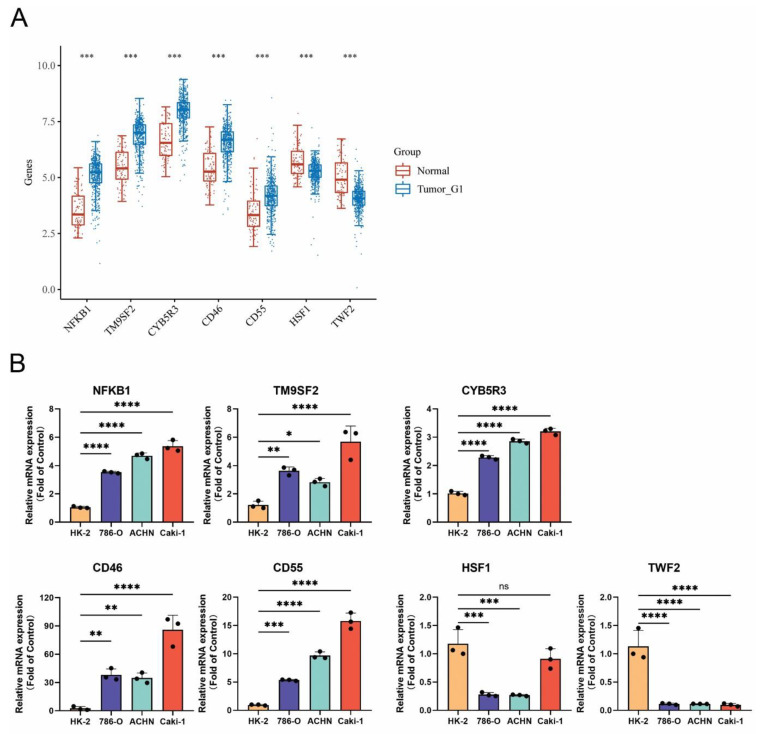
The expression of monkeypox-related genes in KIRC. (**A**) Gene’s transcript level in KIRC based on TCGA and GTEx databases; (**B**) qRT-PCR analysis of MRGs in KIRC (n = 3). *p* < 0.05 was considered significant. * *p* < 0.05, ** *p* < 0.01, *** *p* < 0.001, **** *p* < 0.001, ns: not significant.

In order to increase the reliability of the analyses we performed, we subsequently confirmed the genes expression including *NFKB1*, *TM9SF2*, *CYB5R3*, *CD46*, *CD55*, *HSF1,* and TWF2 by qRT-PCR in kidney renal clear cell carcinoma (KIRC). The HK-2 cell line was used as a control, which is a normal kidney epithelial cell line. The 786-O, ACHN, and Caki-1 cell lines are the kidney renal cell carcinoma cell lines. The gene expression of *NFKB1*, *TM9SF2*, *CYB5R3*, *CD46*, and *CD55* was remarkedly upregulated in KIRC cell lines, while the expression of *HSF1* and *TWF2* was downregulated. The change of the gene expression was consistent with the data analyzed from the TCGA and GTEx databases (Figure 7).

## 4. Discussion

Much remains unknown about the relationship between monkeypox and tumors. In this study, we not only performed a comprehensive and systematic analysis of monkeypox-associated genes in multiple samples from 33 cancer types, but we also comprehensively assessed the roles and mechanisms of MRGs, including clinical features. Monkeypox infection resulted in aberrant expression of MRGs, which was significantly associated with activation of signature-related pathways, clinical survival, tumor immunity, and tumor drug sensitivity. Meanwhile, the prognostic features of monkeypox-related genes were built to be used as biological prognostic markers in tumors. Moreover, this study used consensus clustering to classify UVM into two molecular subtypes based on MRGs, and their overall survival also differed significantly. Following that, immunological and functional analyses were conducted, which revealed the important role of MRGs in the immunological aspects of UVM. As the first analysis of the relationship between monkeypox and pan-cancer, this study explored the correlation of monkeypox-related genes with tumor and immunity, thus elucidating the profound relationship between monkeypox and tumors and providing new strategies for viral therapies targeting tumor immunity.

First, we found that MRGs were differentially expressed in tumors and normal tissues. MRGs tended to be upregulated in tumors and differentially expressed in different tumor subtypes and at different tumor stages. Additionally, survival analysis showed that a high MRGScore was significantly associated with poor tumor prognosis in most cases, and monkeypox may be able to influence tumorigenesis. In addition, genetic analysis revealed that SNV and CNV of MRGs were linked to the survival of tumors. Mutations in monkeypox-related genes were strongly associated with survival, suggesting that mutations may affect tumor prognosis. Therefore, we hypothesize that genetic alterations caused by monkeypox at the genomic level may promote tumor development in some cases.

In pathway analysis, MRGs were identified as key regulators of cancer-associated signaling pathways and were differentially correlated with different cancer-associated signaling pathways. These results suggest that MRGs constitute a network of interactions of cancer-related signaling pathways that may be involved in promoting tumor progression. Meanwhile, this study found that MRGs were correlated with drug sensitivity of anticancer drugs. Among them, 30 drug sensitivities were associated with the expression of MRGs.

To further validate the relationship between monkeypox and tumors and to explore the prognostic value of MRGs in tumor patients, a prognostic risk model of UVM based on MRGs was constructed in this study. The six genes used for risk modeling in this study are closely associated with tumor development and progression. *ADPRH* expression is tightly linked to tumor immune infiltrating cells (TIICs), and its high expression is associated with poor prognosis in glioma [33]. The combination of high *CD8A* and low *HAPLN3* expression can identify subtypes of BLCA patients with good survival rates and help refine the selection of immunotherapy for BLCA patients [26]. *CXCL1*, produced by breast cancer cells, can promote cancer growth and development [27]. *CXCL8* promotes triple-negative breast cancer growth and development, as well as paclitaxel resistance [28]. *IL6* can promote the growth of prostate and rectal cancers [29,30]. *TWF2* deficiency correlates with decreased invasive and migratory capacity of the human hepatocellular carcinoma cell line HUH-7 [34]. The survival analysis in this study showed that the established risk model showed effective predictive performance for the survival of UVM patients. It was associated with elevated CD8^+^ cells and decreased dendritic cells. All of these results show that MRGs play a prognostic role in UVM and that there is a link between monkeypox and tumors.

Throughout, we analyzed the relationship between MRGs and tumor immunity at both the pan-cancer level and UVM. We found that there was a positive correlation between MRGs and immune infiltration in pan-cancer, and there may be some co-expression with most of the immune infiltrating cells. However, the relationship of MRGs in different cell types had a more distinct stratification, which may be related to the different expression and prognostic relevance of MRGs in different tumors. In UVM, MRGs also had a close relationship with immune infiltration, and interestingly, the subgroup with poorer prognosis was instead the one with higher scores of immunity and higher immune infiltration. Nevertheless, as shown by immune checkpoint analysis, immune checkpoint expression was higher in subgroup 1, with poorer prognosis, than in subgroup 2, with better prognosis, and we speculated that MRGs may mediate immune escape of tumors in UVM and even in pan-cancer. In the subsequent investigation, we need to carry out further experimental validation.

The main limitation of this study is that the relationship between monkeypox and cancers has not been studied extensively and there are relatively few research results available as a reference, which may limit the mechanistic elaboration of this study. However, our results showed that the regulation of MRGs existed at all regulatory levels, including genetic and epigenetic alterations, mRNA expression, immune infiltration microenvironment, and pathway correlation. These changes may, in turn, lead to differences in drug efficacy, treatment response, and patient survival. These results explored and showed the connection between monkeypox and tumors, and more importantly, they suggest the feasibility of monkeypox virus-based therapeutic approaches for tumors.

## 5. Summary

In conclusion, we comprehensively elucidated the clinical and immunological profiles of monkeypox-related genes in 33 tumors. Our results showed that the expression of MRGs was connected with tumor prognosis, immunometabolism, and drug sensitivity. A potentially intimate link between monkeypox and tumors exists, indicating that such therapies could be developed if confirmed in the future that monkeypox virus is involved in cancer development.

## Figures and Tables

**Table 1:** Human primers.

Gene	Primers
*CD46*	Forward: AAGTGGTCAAATGTCGATTTCCA
	Reverse: TCGAGGTAAAAACCCTTATCGC
*CD55*	Forward: TTCCAGTCGGTACTGTTGTGG
	Reverse: CCCGGATTAGGGCATGATTTCT
*CYB5R3*	Forward: AAAGTCCAACCCTATCATCAGGA
	Reverse: AAGCGTGCAGAATGTTTGTTC
*HSF1*	Forward: GCACATTCCATGCCCAAGTAT
	Reverse: GGCCTCTCGTCTATGCTCC
*NFKB1*	Forward: AACAGAGAGGATTTCGTTTCCG
	Reverse: TTTGACCTGAGGGTAAGACTTCT
*TM9SF2*	Forward: CGTCAACTTCTGCGACGAAGA
	Reverse: GGCAAAAATCAAACGCTGTGTA
*TWF2*	Forward: AGAAACACCTGTCGTCCTGTG
	Reverse: CACCTCGTTAATGCGGATCTG
β-actin	Forward: CATGTACGTTGCTATCCAGGC
	Reverse: CTCCTTAATGTCACGCACGAT

**Table 2:** The abbreviation of cancer type.

Cancer Type	Abbreviation
acute myeloid leukemia	LAML
adrenocortical carcinoma	ACC
uroepithelial carcinoma of the bladder	BLCA
invasive breast cancer	BRCA
squamous cell carcinoma of the cervix and endocervical adenocarcinoma	CESC
cholangiocarcinoma	CHOL
colonic adenocarcinoma	COAD
esophageal cancer	ESCA
glioblastoma multiforme	GBM
head and neck squamous cell carcinoma	HNSC
renal suspicious cell carcinoma	KICH
renal clear cell carcinoma	KIRC
renal papillary cell carcinoma	KIRP
low grade glioma	LGG
hepatocellular carcinoma	LIHC
lung adenocarcinoma	LUAD
lung squamous cell carcinoma	LUSC
lymphoid neoplasm spreading large b-cell lymphoma	DLBC
mesothelioma	MESO
ovarian plasmacytoid cystic adenocarcinoma	OV
pancreatic adenocarcinoma	PAAD
pheochromocytoma and paraganglioma	PCPG
prostate adenocarcinoma	PRAD
rectal adenocarcinoma	READ
sarcoma	SARC
cutaneous melanoma	SKCM
gastric adenocarcinoma	STAD
testicular germ cell tumor	TGCT
thymoma	THYM
thyroid cancer	THCA
uterine carcinosarcoma	UCS
endometrial cancer of the uterine corpus	UCEC
uveal melanoma	UVM

**Table 3:** The abbreviations of monkeypox-related genes (MRGs).

Gene	Abbreviation
ADP-Ribosylarginine Hydrolase	*ADPRH*
BMS1 Pseudogene 20	*BMS1P20*
Complement C4A	*C4A*
Complement C4B	*C4B*
Chemokine (C-C motif) ligand 26	*CCL26*
Cluster of differentiation 4	*CD4*
CD46 molecule	*CD46*
CD55 molecule	*CD55*
CD8a Molecule	*CD8A*
Complement Factor H	*CFH*
Complement C3d Receptor 1	*CR1*
Complement C3d Receptor 2	*CR2*
Crystallin Gamma C	*CRYGC*
C-X-C Motif Chemokine Ligand 1	*CXCL1*
C-X-C Motif Chemokine Ligand 8	*CXCL8*
Cytochrome B5 Reductase 3	*CYB5R3*
Exostosin Glycosyltransferase 1	*EXT1*
Heat Shock Transcription Factor 1	*HSF1*
Interferon Alpha 1	*IFNA1*
Interleukin 6	*IL6*
Interferon Regulatory Factor 3	*IRF3*
Killer Cell Lectin-Like Receptor C3	*KLRC3*
Killer Cell Lectin-Like Receptor K1	*KLRK1*
DNA Ligase 4	*LIG4*
MX Dynamin-Like GTPase 1	*MX1*
Nuclear Factor Kappa B Subunit 1	*NFKB1*
Transmembrane 9 Superfamily Member 2	*TM9SF2*
Transmembrane P24 Trafficking Protein 10	*TMED10*
Twinfilin Actin-Binding Protein 2	*TWF2*
Zinc Finger Protein 846	*ZNF846*
Olfactory Receptor Family 10 Subfamily G Member 6	*OR10G6*

## Data Availability

The data that support the findings of this study are available on request from the corresponding author. The data are not publicly available due to privacy or ethical restrictions.
